# Aging-Induced QT Prolongation as a Potential Contributor to Longevity

**DOI:** 10.3390/jcdd13020086

**Published:** 2026-02-09

**Authors:** Simon W. Rabkin

**Affiliations:** Division of Cardiology, University of British Columbia, Vancouver, BC V5Z 1M9, Canada; simon.rabkin@ubc.ca

**Keywords:** QT interval, aging, sudden cardiac death, transmembrane cardiac ion flux, epigenetics

## Abstract

The objective of this paper was to review the possibility that the QT interval may be a marker of adult human longevity or life expectancy. Following a literature review, data supporting this possibility was assembled and consists of the following. First, in adults, QT interval increases with increasing age. This is analogous to aging-induced hypertension and diabetes mellitus, both of which are associated with shorter longevity. Second, older persons frequently die suddenly regardless of whether or not they have chronic illnesses for which death is expected. Third, longer QTintervals are associated with increased probability of sudden death. Fourth, patients with two conditions associated with accelerated brain aging, namely dementia and Parkinson’s disease, show longer QTcs than age-matched controls. Both of these conditions are associated with sudden cardiac death. Fifth, aging processes may affect the molecular determinants of the QT interval, alter heart composition with increased myocardial fibrosis, or alter the amount of sympathetic and parasympathetic tone, any or all of which can alter myocardial repolarization and the duration of the QTc. Sixth, considering the molecular determinants of the QT interval in the aging heart, which has longer transmembrane action potentials, several factors can account for this change, including changes in late inward Na^+^ current (I_NaL_), I_Kr_, I_ca_, I_to_, and K_ATP_ channels. Transgenic mice overexpressing the Kir6.1 subunit of a K_ATP_ channel show a prolonged QT interval and reduced longevity, with animals appearing to die suddenly. Seventh, chronic kidney disease, which is associated with a reduced lifespan, is associated with reduced expression of the anti-aging factor Klotho and Klotho-deficient mice have a prolonged QTc and a reduced lifespan. Taken together, there is a cogent case for factors that increase action potential duration in the aging heart, as recognized by increased QTc, to act in concert with other factors to produce fatal arrhythmias leading to sudden cardiac death and shortened longevity.

## 1. Introduction

The study of longevity, the scientific investigation of the limitations on individual survival, aims at retarding the aging process, which is a complex amalgamation of genetic, environmental, metabolic, and cellular factors [1,2]. These studies aim to identify potentially reversible factors that are determinants of mortality, as well as morbidity, associated with aging [1]. One of the types of limitations on longevity is sudden death, which is usually cardiac in origin. Sudden death is a common condition [3,4,5,6]. Older persons frequently die suddenly regardless of whether or not they have chronic illnesses for which death is expected, but its occurrence is sudden in nature [7,8]. It has been estimated that the incidence of sudden death for 80-year-old men is approximately seven times greater than that for men aged 40, and the incidence of sudden death in a woman over 79 years is approximately 40-fold greater than for women less than 45 years of age [8,9,10]. Sudden death, when cardiac in origin, is produced by cardiac arrhythmias that include ventricular tachycardia, ventricular fibrillation, pulseless electric activity or asystole [5]. Although implantable cardioverter–defibrillators (ICD) can be effective in the prevention of sudden cardiac death, they are likely to benefit only a small population at very high risk for sudden death [5]. Prophylactic ICD therapy, however, appears to be less beneficial in older compared to younger individuals [11]. Also, the cost-effectiveness thresholds for ICD are higher in elderly populations than in younger patients, as life expectancy is shorter in older adults [8].

Identifying factors that may lead to a reduction in longevity, specifically those leading to sudden cardiac death in older individuals and that may be subject to modification by medical therapy, is an important goal. The QT interval on the electrocardiogram, a measure of cardiac repolarization, has been linked to fatal ventricular arrhythmias in inherited conditions and drug toxicity [6,12]. Whether the QT interval should be considered a determinant of longevity has not been explored in depth. The objective of this review is to examine the hypothesis that the QT interval is an indicator of or determinant of life expectancy or longevity. To our knowledge there has not been a previous review on this subject. To address this question, a review process was established (Supplementary material).

## 2. QT Interval and Aging

QT interval, traditionally measured from the onset of the QRS complex to the end of the T wave, consists of two components: the QRS complex, which reflects depolarization within the His–Purkinje system and the ventricles, and the JT interval, which reflects the duration of ventricular repolarization [13,14]. It is the assessment of ventricular repolarization that has attracted attention, because measuring the QT interval is the easiest way to assess ventricular repolarization on the surface ECG.

**Figure 1:**
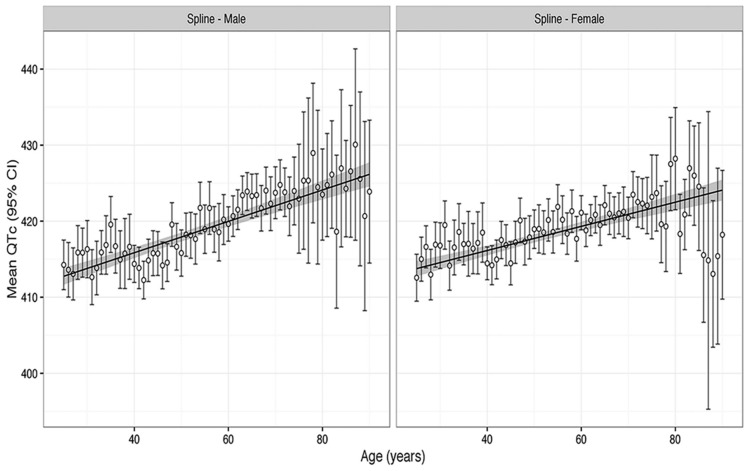
The relationship between QT interval and heart rate for mean spline QTc, with 95% confidence intervals in different age groups from the US NHANES survery for men (**left**) and women (**right**). The linear regression (line) of age on spline QTc is plotted with the 95% confidence interval. The figure (with permission) is from [15].

Because the QT interval varies with heart rate, it is crucially important to utilize the best QT–heart rate adjustment formula or so-called correction factor (QTc). We have shown that a formula with a spline function is the best formula to correct for the effect of heart rate on the QT interval [15]. This approach, developed with data from over 12,000 individuals, clearly demonstrates an increasing QT interval with increasing age, i.e., a significant relationship between age and QT interval (Figure 1).

**Figure 2:**
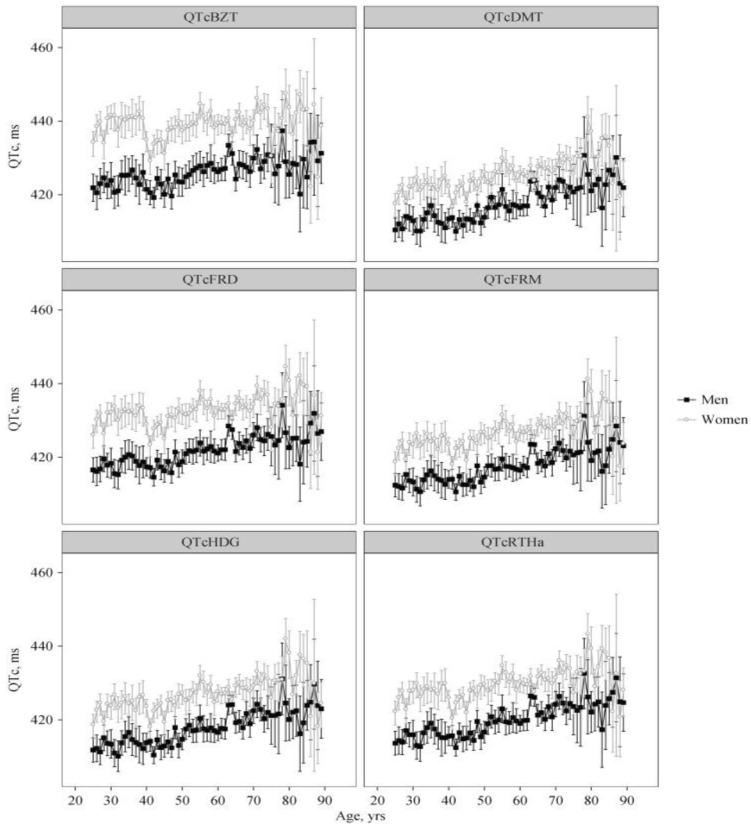
The relationship between QT interval and heart rate for various other QT–heart rate adjustment formulae. QT–heart rate adjustment formulae, specifically the QTc: proposed by Bazett (QTcBZT), Fridericia (QTcFRD), Hodges (QTcHDG), Dmitrienko (QTcDMT), Rautaharju (QTcRTHa), and Framingham (QTcFRM). The figure (with permission) is from [16].

An in-depth analysis of the relationship between QT interval and an individual’s age used six different QT–heart rate adjustment formulae, specifically the QTc formulae proposed by Bazett (QTcBZT), Fridericia, Rautaharju, Framingham, Hodges, and Dmitrienko [16]. The QTc was strongly related to age for all formulae except for QTcBZT for women (Figure 2).

The finding that QTc increased with age was significant even if the relationship was approximated by a linear or non-linear (quadratic or cubic spline) model [16]. There was a greater proportion of longer QTc in individuals in the very old ages: ages 80 years and above [16]. The wider confidence intervals in the older age group (Figure 1) reflect the smaller sample size of older patients [15]. The smaller sample size at older age in women likely accounts for the reduction in QT interval in the oldest group. Interestingly, the QTc increased more dramatically with age in men than women [16]. Our analysis in a large population is supported by several other studies. In a small sample (151 persons), Reardon et al. found a significant correlation between aging and prolonged QTc in both women and men [17]. Longitudinal data also support the concept that aging is associated with increasing QT duration. Su et al. followed a group of 151 individuals with an average age of 73 years and reported that the QTc interval increased significantly during a 4-year follow-up [18].

QTc increases with age due to a combination of factors. Aging processes may affect the molecular determinants of the QT interval (discussed below) or alter the myocardium with increased myocardial fibrosis [19], or the QT interval may be an indicator of biological aging [20]. Aging is also associated with alterations in the amount of sympathetic and parasympathetic tone [21], which can alter myocardial repolarization and the duration of the QTc [14].

Another interesting observation is related to the presence of a longer QT interval in individuals with Parkinson’s disease [22]. The prevalence of Parkinson’s disease increases with aging [23,24]. Compared to individuals 40 to 49 years of age, the prevalence of Parkinson’s disease is 10-fold greater in those aged 65 to 74 years, and Parkinson’s disease prevalence is over 45 times greater for those over the age of 80 years [23,24]. The QT interval is significantly longer in patients with Parkinson’s disease compared to controls with an odds ratio over two [22]. A similar observation is present for individuals with dementia, another age-associated condition, in which longer QTcs are observed in older persons with dementia compared to controls [25]. Thus, the aging-brain-associated conditions of Parkinson’s disease and dementia are associated with aging-induced QT prolongation.

Variation in the QT interval, i.e., the difference between the longest and shortest QT interval on a 12-lead ECG, has been labeled QT dispersion, because there is a significant correlation between variations in QT interval and the dispersion of repolarization as determined by the dispersion of recovery time and action potential duration in the isolated heart [26]. The ability of QT variability to reflect QT dispersion has been criticized, appropriately, because of the weak association between this measurement and more accurate evaluations of QT heterogeneity [27,28]. However, some patient population studies found that increased QT variability (‘dispersion’) was associated with subsequent death from cardiac disease [29,30]. Interestingly, QT variability is significantly increased in the 85 and older age group compared to the 75-to 84-year age group and especially compared to those 65 to 74 years of age [31]. These data raise the possibility that aging increases QT variability, perhaps reflective of ventricular repolarization, which might be a contributing factor leading to cardiac mortality [31].

## 3. QT Interval and Subsequent Cardiac Death

QT interval prolongation has been linked to sudden cardiac death for a long time [32,33,34]. The suggestion that QT interval length is causally linked to sudden cardiac death is supported by the presence of genetic polymorphisms that increase QT and produce cardiac sudden death [35]. In the general populations, several epidemiologic studies have linked QT duration with subsequent mortality from cardiac disease or sudden cardiac death [29,33,36,37,38,39]. Prolongation of cardiac repolarization, which is detected on the electrocardiogram as prolongation of the QT interval, has been proposed to be a substrate for potentially fatal cardiac arrhythmias [40,41,42]. In a literature review of studies from 1966 to 1999, Bednar et al. concluded that most but not all general population studies found that a prolonged QTc was associated with increased risk for cardiac death [13]. In some of these studies the increased risk did not change after adjustment for potential confounders, including history of myocardial infarction, hypertension, and diabetes mellitus [39], suggesting that the relationship of QT interval to subsequent cardiac death is independent of the presence of cardiac disease [37]. Subsequent to the review by Bednar et al., a meta-analysis of population studies found a consistent association between prolonged QT interval and increased risk of sudden cardiac death, as well as death from all cardiac causes [42].

In the LIFE study (Losartan Intervention for Endpoint Reduction in Hypertension), of hypertensive patients identified as being at high risk by the presence of ECG LVH, their baseline QT interval further stratified the risk of mortality despite an intervention to effectively lower blood pressure [43]. In a multivariate analysis, adjusting each ECG measure for covariates (gender, baseline systolic and diastolic blood pressure, treatment arm [losartan or atenolol], baseline Framingham risk score, Sokolow–Lyon voltage and Cornell voltage duration product, QT interval remained a significant predictor of mortality [43].

Considering that aging is associated with prolongation of the QT interval and increased QTc is associated with cardiac death, it is reasonable to propose that aging-induced QTc prolongation is a factor accounting for cardiac mortality, and thus longevity, in human aging. It is interesting to note that in the age-associated condition of Parkinson disease, sudden cardiac death is the second most common cause of death, after pneumonia [44].

The ability of a cardiac factor derived from the ECG to act as a determinant of longevity is not a new concept, but one that has received recent attention [45]. The novelty of the present study is that it focusses on a single ECG variable that is linked to cardiac death. The association of longer QT interval and cardiac death should not be considered the only cause of sudden death in older persons, as myocardial ischemia, heart block, and other established causes of sudden death are still operative and cause cardiac death in older individuals.

One potential critique about linking age-associated QTc and reduced longevity is that it might appear that as people live longer, their QT interval will also be longer, which is the reverse of our suggestion. The data support our contention because individuals who do not experience a shortened longevity, namely those who live to be over 100 years of age, do not have an increased QTc [46]. A similar critique is that physiological factors such as blood pressure and glucose levels also increase with aging, suggesting that it is difficult to conclude that these changes are potential contributors to longevity. This objection is not valid because aging is associated with an increased prevalence of elevated blood pressure (hypertension), which in turn is an independent predictor of adverse cardiovascular outcomes, such as myocardial infarction, cognitive decline, stroke, and kidney diseases [47]. Individuals who do not manifest aging-induced hypertension have a low risk of atherosclerotic cardiovascular disease [48]. Similarly, aging-induced alterations in glucose metabolism are a component of the increased prevalence of diabetes mellitus in older individuals and represent a group at high risk of complications and adverse geriatric syndromes that require vigorous interventions [49,50].

## 4. Mechanisms by Which QT Prolongation Is Associated with Cardiac Sudden Death

The link between QT interval prolongation and cardiac sudden death has been explained by a number of investigators [51,52,53]. Prolonged QT interval reflects a prolongation of cardiac transmembrane action potential that leads to an increased probability of L-type calcium channels reopening that produces early afterdepolarization [52]. Early afterdepolarizations (EADs) produce triggered activity that can initiate ventricular arrhythmias [54]. In addition, if the action potential duration is not homogenous throughout the myocardium, there can be areas of unidirectional block and electrical (rotor) instability leading to polymorphic ventricular tachycardia [51]. The increased susceptibility to ventricular tachycardia, as manifested by circus-type or spiral wave re-entry, occurs in part from increased transmural dispersion of repolarization [53].

### 4.1. Aging and Ventricular Arrhythmias

Ventricular ectopy is a sign that the myocardium can generate ventricular arrhythmias and these arrhythmias are more common in older individuals [55]. The prevalence of ventricular arrhythmias increases with aging. In a population sample of 1512 individuals over the age of 65 years, brief episodes of ventricular tachycardia (three or more consecutive ventricular depolarizations) were detected in 4.3% of women and 10.3% of men [55]. This observation has also been noted in aging mice that manifest an increased presence of one or multiple premature ventricular complexes [56]. In patients with ventricular tachycardia, increasing age is associated with higher mortality and compared to middle-aged individuals, these older persons had a significantly higher long-term mortality [57].

Aged hearts are highly susceptible to early afterdepolarization-mediated ventricular fibrillation [58]. Experimentally, susceptibility to ventricular fibrillation in response to beta-adrenergic stimulation after ischemia/reperfusion injury is many-fold higher in older rat hearts compared to younger hearts [59]. Older hearts fail to show, to the same extent, the shortening of action potential duration in response to isoproterenol [59].

Potential explanations for the association of aging with an increased prevalence of ventricular arrhythmia include several different mechanisms. First, a greater sensitivity of ryanodine receptors (RyRs) to diastolic Ca^2+^ and the greater tendency for spontaneous opening of RyRs, which are the cause of premature ventricular complexes has been suggested [60]. Although SERCA2a mRNA, protein and activity levels decline with age implying a reduced rate of Ca^2^^+^ uptake by the SR, it has been suggested that age has no effect on expression of RyR2 protein in hearts [61]. Second, aging is also associated with degeneration of the cardiac contractile elements and conduction system which are replaced by fibrosis [62]. In addition, interstitial fibrosis develops with aging independently of cardiomyocyte loss (death) and has been ascribed to the aging process [63,64]. Cardiac fibrosis leads to heterogeneity of electrical conduction which in turn predisposes individuals to cardiac arrhythmias [62]. Premature ventricular ectopy is more likely to lead to ventricular tachycardia or fibrillation in the presence of structural changes in the heart, such as myocardial fibrosis Third, the aging heart has a greater likelihood of developing ventricular tachycardia or ventricular fibrillation when exposed to catecholamine stress or oxidative stressors [61]. Compounding the increased sensitivity is the increase in oxidative stressors that are produced by the mitochondria in the aging heart [61].

A different kind of link between aging, increased QT interval, and longevity involves the anti-aging factor Klotho. There are several different Klotho proteins, the expression of which varies in different organs, and they are a recognized anti-aging factor [65,66]. Klotho-deficient mice have a prolonged QTc and a reduced lifespan [67]. Chronic kidney disease, which is associated with a reduced lifespan, is associated with reduced expression of Klotho [67]. In an animal model of chronic kidney disease, Klotho protected against the prolonged QT interval induced by uremia and promoted by fibroblast growth factor 23 [67].

Another aspect worthy of discussion is the complex pathophysiology of aging-associated cardiac hypertrophy and fibrosis. Various factors stimulate fibroblasts to become myofibroblasts that secrete large amounts of extracellular matrix leading to pathological fibrosis, which is a major risk factor for the development of cardiac arrhythmias and heart failure [68]. Transient receptor potential (TRP) channels, which regulate the cardiac hypertrophic response, appear to coordinate signaling within local domains or through direct interaction with Ca^2+^-dependent regulatory proteins in coordination with signaling through effectors such as calcineurin and the nuclear factor of activated T cells [69]. Transient receptor potential canonical (TRPC) channels and transient receptor potential of the melastain subfamily proteins (TRPM7) are involved in the pathogenesis of cardiac hypertrophy and cardiac arrhythmias [70,71]. These channels can be activated by reactive oxygen/nitrogen species [72,73] and reactive oxygen/nitrogen species have been implicated in processes that curtail longevity, although the relationship is complex [74].

This complexity is further accentuated by the relationships between the general cellular senescence phenomena and specific cardiac changes, as reflected in the transcriptomic profile, which is influenced by other factors, producing age-dependent QT interval changes. Within this constellation of factors, aging increases the prevalence of ventricular arrhythmias, and in the presence of a longer QT interval the combination is more likely to induce potentially fatal arrhythmias of ventricular tachycardia or fibrillation.

### 4.2. The Underlying Mechanism by Which Aging Induces QT Prolongation

QTc increases with age potentially due to a combination of factors. Aging processes may affect the molecular determinants of the QT interval or alter the myocardium because of an increased myocardial fibrosis [19]. Aging is also associated with alterations in the amount of sympathetic and parasympathetic tone [21], which can alter myocardial repolarization and the duration of the QTc [14].

An intriguing speculation is that genetic and/or epigenetic factors account for the relationship between QT interval and longevity. Variants in the KCNQ1 and KCNH2 genes that are associated with Long QT syndromes were associated with a 23-fold increased odds of marked corrected QT interval prolongation in a population-based study of 29,000 individuals (in the UK Biobank study) [75]. These gene variants may not be manifested until old age and may be responsible for death in older persons. Another speculative possibility is the epigenetic changes that occur with aging. Epigenetic changes include: DNA methylation, histone modification, chromatin remodeling, non-coding RNA (ncRNA) regulation, and RNA modification [76], which all participate in aging and can be involved in age-related conditions and diseases [77]. If epigenetic factors are involved, small-molecule-based therapies and reprogramming strategies that are being developed to limit or prevent epigenetic processes may be useful in blocking the pathways leading to aging-induced QT prolongation [77].

Another postulated mechanism is that aging produces cardiomyocyte cell death leading to the tissue response of fibrosis, as a method of repair, because of the proliferation of fibroblasts and the differentiation of fibroblasts to myofibroblasts. Myofibroblasts are a source of proinflammatory cytokines, IL-1β, IL-6, and TNFα [78]. The whole-body deterioration with aging has been labeled as frailty which is characterized as wasting and weakness that correlates with dysregulation of the immune, endocrine, and neurohumeral systems, as manifest in part by increased inflammatory cytokines [20,79]. Thus, cardiac aging with an increased expression of cytokines may be part of the aging process of the entire body.

### 4.3. Increased Action Potential Duration from Alteration of the Molecular Determinants of QT Interval

Cellular and molecular mechanistic evidence derives from animal cardiac electrophysiology that differs from humans. With this caveat, the data provides insight into the possible explanations for aging-induced lengthening of the QT interval. The cardiac action potential is longer in the heart and in cardiomyocytes from older compared to younger guinea pigs, mice, and rats [56,80,81,82,83,84]. Increased cardiac action potential duration was also found in a model of senescence in cardiomyocytes induced by doxorubicin [85]. This change in action potential duration is consistent with and reflective of an age-related increase in QT interval as action potential duration is responsible, in part, for the duration of the QT interval [13].

Action potential duration is determined by a variety of different transmembrane inward and outward currents [13]. One of these factors that has attracted recent attention is the late inward Na^+^ current (I_NaL_), a component of the fast Na^+^ current of cardiac myocytes [86]. Several lines of evidence link I_NaL_ to aging-induced prolongation of the QT interval and cardiac action potential duration. First, the amplitude of I_NaL_ in cardiomyocytes from older guinea pigs is much greater than I_NaL_ in cardiomyocytes from younger guinea pigs [83]. Second, and importantly, the age-related prolongation of the QTc or cardiac action potential was reversed by I_NaL_ inhibitors [56,83]. Inhibition of I_NaL_ shortens the QT interval in older but not younger mice [56]. Third, in a model of senescence in cardiomyocytes, there is an enhancement of I_NaL_ resulting in a prolonged QTc interval [85]. Fourth, mice with I_NaL_ gain-of-function mutation accentuate the changes with aging, while animals with I_NaL_ loss-of-function mutation are protected from the age-related changes in ventricular repolarization and are insensitive to the corrective effects of I_NaL_ inhibition [56]. Interestingly, ranolazine prevents the I_NaL_ enhancement-blunted myocardial remodeling in a model of pulmonary hypertension [87].

Nav1.5 (previously referred to as SCN5A) is involved with sodium influx in cardiomyocytes. The phosphorylation level of Nav1.5 at Ser571 is enhanced in the myocardium of older compared to younger mice while the expression of Nav1.5 is unchanged [56]. These data suggest that aging-induced factors alter phosphorylation of key components of cardiac currents influencing cardiac transmembrane action potentials.

A potential mechanism whereby aging can affect action potential duration is from reactive oxygen generation which can be increased in aging. Free radicals such as hydrogen peroxide prolong action potential duration and enhance I_NaL_ by slowing its kinetics of inactivation [88]. Blockade of the late sodium current reduces hydrogen peroxide-induced arrhythmogenic activity and contractile dysfunction [89]. These actions of hydrogen peroxide on old myocytes are attenuated by I_NaL_ inhibition [83]. Cardiomyocyte I_NaL_ from older guinea pigs can also be inhibited by a Nav1.5 channel blocker or a Ca^2+^/calmodulin-dependent protein kinase (CaMKII) blocker [83]. The role of calmodulin inhibition is consistent with data that phosphorylation by CaMKII of sodium channels enhances I_NaL_ in cardiac myocytes, supporting the contention that the increased production of reactive oxygen species in aging hearts enhances CaMKII activation, which in turn increases I_NaL_ [83]. Senescence or biological aging in cardiomyocytes is associated with increased oxidative stress, alteration in mitochondrial morphology, and depolarized mitochondrial membrane potential [85].

The rate of increase in QTc in older men is greater than in women [16]. This observation may relate to the loss of testosterone in older men. Testosterone deficiency in animals is associated with prolonged repolarization, abnormal electrical activity, larger late sodium currents, and increased expression of NaV 1.8 sodium channels in cardiomyocytes [90].

I_Kr_ changes with aging are also responsible for prolongation of the cardiac action potential but likely to a lesser extent. Induced senescence in cardiomyocytes shows an increase in action potential duration as well as a significant downregulation of I_Kr_ density and a reduction in HERG channel protein expression [85]. There is a significant inverse correlation between KCNH2 expression in atria removed at time of cardiac surgery and the age of the patient [85]. In contrast to these findings, other investigators reported that I_Kr_ did not appear to be a significant factor in the aging guinea pig heart [83].

Other currents may be involved in the increase in action potential in the aging heart but not to a significant extent. I_Ca_ was not found to be abnormal in one study [83], but I_Ca_ inactivation was reported to be slowed with aging in another study [91]. A minor role for calcium currents is supported by the absence of changes in Ca^2+^ influx through L-type Ca^2+^ channel and peak I_CaL_ density in senescence-induced cardiomyocytes [85]. A reduction in I_to_ channel density with aging has been reported [91]. I_Ks_ does not appear to be a significant factor in aging, although a moderate decrease in I_K1_ in old myocytes is possible [83]. Whole-cell K_ATP_ channel current density is reduced in ventricular myocytes from aged rat and mouse hearts due to an enhancement of the inhibitory effect of cytosolic ATP on channel activity [81]. The K_ATP_ channel is a heteromeric protein of four pore-forming Kir6 with other components, and downregulation of the K_ATP_ channel in cardiomyocytes occurs in cardiomyocytes from transgenic mice overexpressing the Kir6.1 subunit [92]. This transgenetic mouse strain shows a prolonged QT interval [92,93]. It also showed a reduced longevity with animals appearing to die suddenly, presumably from cardiac arrhythmias [93].

## 5. Study Limitations

The challenges with the construct that the QT interval relates to longevity is the current lack of traditional scientific proof based on experimentation in human aging. Once sufficient evidence is accumulated, a randomized clinical trial of an agent that shortens QT interval with a low risk of adverse effects can be conducted in an older population with the outcome of prevention of cardiac death. The current study sets the foundation for conducting further research. Another issue is that while significantly prolonged QT intervals can provoke arrhythmia, so too can very short QT intervals. However, a short QT interval should not be relevant for this concept because it is uncommon, especially in older individuals, and is due to specific metabolic or rare genetic abnormalities [94]. Another issue is that age-related QT interval prolongation is shorter than the QT ranges associated with the risk of sudden death in long QT syndrome-related disorders [35]. It is instructive, however, to examine the epidemiologic or population data on QT interval and mortality. Zhang et al. conducted a meta-analysis on 23 observational and epidemiologic studies with over 130,000 individuals [41]. The pooled relative risk estimates comparing the highest with the lowest categories of QT interval length were 1.35 for total mortality, 1.51 for cardiovascular mortality, 1.71 for coronary heart disease mortality, and 1.44 for sudden cardiac death [42]. The highest QT interval was often a QT interval over 440 ms (usually calculated by the Bazett formula) [42], which is much shorter than the QT interval in inherited QT prolongation syndromes associated with sudden death. In some cohorts such as the Rotterdam study, a QTc over 440 ms was associated with an over two-fold increased risk of sudden cardiac death after adjustment for age and sex [40].

The most common causes of QT prolongation are electrolyte abnormalities, medications, and comorbidities such as thyroid or cardiac disease. QT prolongation in older adults may be driven by medications which are more commonly prescribed for older individuals, but this is not likely to be responsible for the increase in QT interval across age groups. Drugs that have the potential to prolong the QT interval are only taken by a small percentage of older persons [95,96]. Furthermore, the use of potentially QT-prolonging drugs is more common in hospitalized older persons [95,96], who are not usually included in population studies that define the QT interval across different age groups.

QT prolongation in older adults may also occur because of non-cardiac diseases or comorbidity, which are more prevalent in older populations [22,25]. However, the increased risk from longer QTc intervals is not changed after adjustment for potential confounders, including a history of myocardial infarction, hypertension, and diabetes mellitus [39], suggesting that the relationship of QT interval to subsequent cardiac death is independent of the presence of cardiac disease [37].

In clinical practice or on an individual patient perspective, a level of QT prolongation has been defined by a predetermined corrected QT interval that is longer than the mean. QTc increment with aging, raising the suggestion that these small increases are sufficient to trigger lethal arrhythmias. In clinical practice there is agreement that a QT interval of at least 500 milliseconds correlates with a higher risk of potentially fatal ventricular tachycardia and there is no established threshold below which prolongation of the QT interval is considered free of proarrhythmic risk [97,98,99]. Population studies found that individuals with QT intervals in the highest percentile (over 75th) have a significant 1.6-fold increase in all-cause mortality, for men and women, when QT (using Fridericia adjustment) is 426 ms or greater for men and 432 ms or greater for women [39] or when using the Bazett formula the 75th percentile is 437 for men and 446 for women with an overall 1.8-fold increased risk of all-cause mortality [39]. Data from inherited prolonged QT syndromes and drug-induced QT prolongation has suggested that every 10 ms increase in QTc produces a significant increase in the risk of ventricular tachycardia or Torsade de Pointe [97,98,99].

## 6. Future Research

A reasonable suggestion to test this thesis is with agents that shorten the QT interval [100] in a population of older individuals. Possible candidate molecules are those that act to provide a small degree of inhibition of I_NaL_, to produce a QTc found in younger individuals. I_NaL_-inhibiting drugs have been demonstrated to be potential antiarrhythmic agents, based on experimental, preclinical, and clinical trial data [100]. Some are clinically available but have not been tested in older individuals for the prevention of death and extension of longevity.

## 7. Conclusions

**Figure 3:**
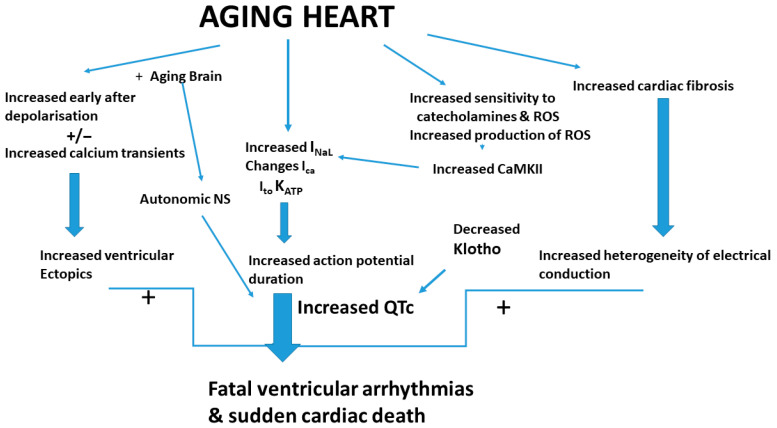
Graphic incoporating some of the changes in the aging heart that link QT interval to sudden cardiac death and other factors that facilitate sudden death along with the changes in QT interval.

In summary, the data show that aging is associated with an increase in QT duration; epidemiologic studies show that a longer QT interval is associated with sudden cardiac death and clinical studies show that non-cardiac conditions associated with prolonged QT intervals have an increased occurrence of cardiac death. Factors leading to shortened longevity such as deficiency of the anti-aging factor Klotho is associated with QT prolongation. Scientific experimental studies report cardiac action potential is longer in the heart and in cardiomyocytes from older compared to younger guinea pigs, mice, and rats, and aged hearts are more susceptible to cardiac arrhythmias. In aged hearts and in a model of senescence in cardiomyocytes, there is an enhancement of I_NaL_ resulting in a prolonged QTc interval. Importantly, aging is associated with alterations in a K_ATP_ channel and transgenic animals overexpressing one of the pore-forming units of a K_ATP_ channel have a prolonged QT and a shorter longevity marked by sudden death. Aging-associated prolongation of QTc is due to a constellation of factors, due to aging that directly impact the currents and channels responsible for the action potential as well as the impact of the aging brain on the heart (Figure 3).

## Data Availability

No new data were created or analyzed in this study.

## Supplementary Materials

The following supporting information can be downloaded at https://www.mdpi.com/article/10.3390/jcdd13020086/s1.

## Abbreviations

The following abbreviations are used in this manuscript:

RyRs	Ryanodine Receptors
I_NaL_	Late Inward Na^+^ Current
K_ATP_ channels	
CaMKI	Ca^2+^/Calmodulin-Dependent Protein Kinase
EAD	Early Afterdepolarizations
