# Supplementary material for: Aging-Induced QT Prolongation as a Potential Contributor to Longevity
Source: J Cardiovasc Dev Dis. 2026 Feb 9;13(2):86. doi: 10.3390/jcdd13020086 (PMC12942042; doi:10.3390/jcdd13020086)
Supplement: Supplementary file 1 [file jcdd-13-00086-s001.zip › jcdd-4033562-supplementary.pdf]

Aging-Induced QT Prolongation as a Potential Contributor to Longevity

Simon W Rabkin

Methods

Search strategy and study selection

This systematic review was conducted according to the Preferred Reporting Items for Systematic Reviews. The review protocol was not previously published. A literature search was conducted across PubMed and MEDLINE from database inception to December 30, 2025, assessing QT interval and longevity or life expectancy. Abstracts and full texts were screened and assessed.

The inclusion and exclusion criteria were defined. Inclusion criteria were used: (i) adults (age ≥ 18 years for clinical studies (II) cellular or molecular mechanisms. Exclusion criteria included pediatric (age < 18) for clinical studies, genetic or inherited conditions editorials, commentaries, conference abstracts, reviews, or non-English studies.

Risk of bias assessment could not be performed because most of the studies were basic science investigations

PUBMED

Longevity	73,401
Life expectancy	8169
QT interval	16,690
Longevity and QT interval	11
Life expectancy and QT interval	22

MEDLINE

Longevity	12,873
Life expectancy	46,143
QT interval	11,663
Longevity and QT interval	2
Life expectancy and QT interval	4
